# A pilot study of intralesional methotrexate injections versus triamcinolone acetonide in patients affected by nail matrix psoriasis

**DOI:** 10.1111/ced.15110

**Published:** 2022-02-18

**Authors:** Michela Starace, Aurora Alessandrini, Matilde Iorizzo, Ambra D'Altobrando, Tiziano Ferrari, Francesca Bruni, Bianca Maria Piraccini

**Affiliations:** ^1^ Dermatology Unit, IRCCS Policlinico Sant'Orsola, Department of Experimental Diagnostic and Specialty Medicine (DIMES) Alma Mater Studiorum University of Bologna Bologna Italy; ^2^ Private Dermatology Practice Lugano and Bellinzona Switzerland; ^3^ Dermatology Unit, AUSL Romagna Ravenna Italy

## Abstract

Nail disorders in general are difficult to treat and often frustrating, and this is also the case with nail psoriasis, especially when it is limited to the nails, and not affecting joints. The quality of life of patients with nail psoriasis is negatively affected, owing to the chronic course of the disease and frequent relapses. The purpose of this study was to compare treatment response and maintenance of response during follow‐up of 12 patients with nail matrix psoriasis limited to a few nails, who were treated with intralesional injections of either methotrexate (MTX) 25 mg/mL or triamcinolone acetonide 10 mg/mL. Patients were treated every 6 weeks for 24 weeks and followed up for 6 months. Photographic documentation and assessment by Nail Psoriasis Severity Index were performed during each treatment session and at each follow‐up visit. At the end of the four treatment sessions, all patients had improvement of their disease, which continued during follow‐up, especially for the MTX‐treated group.

## Graphical abstract

The purpose of this study was to compare the response to treatment and follow‐up of 12 patients with nail matrix psoriasis, limited to few nails, treated with intralesional injections of methotrexate 25 mg/mL or triamcinolone acetonide 10 mg/mL.

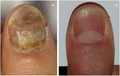

Nail disorders in general are difficult to treat and often frustrating, and this is also the case with nail psoriasis, especially when it is limited to the nails. Based on recent expert consensus,^1^ it appears that intralesional injections of triamcinolone acetonide (TA) are an excellent treatment for nail psoriasis because the drug is deposited where the inflammatory infiltrate occurs, without the risk of systemic adverse events (AEs). Injections are particularly useful to treat psoriasis of the nail matrix, as this area is difficult to reach with topical agents. This area can also be easily treated without digital anaesthesia compared with the nail‐bed area. However, local AEs can occur with TA, thus there have been a number of studies on the efficacy and safety of other active treatments injected into the nail unit.^2^, ^3^, ^4^, ^5^, ^6^, ^7^ Both matrix and bed have been injected with methotrexate (MTX), but only one study has compared MTX with other active treatments such as TA and ciclosporin (CsA); good outcomes were reported for TA and MTX, which both appear to be better than ciclosporin.^4^

We report our own experience with intralesional injections of MTX compared with TA in adult patients with nail‐matrix psoriasis, but with a more severe clinical presentation than patients previously reported in the literature.

## Report

The study was approved by the University of Bologna (Italy) and all patients provided signed, written informed consent before participating in the study.

Inclusion criteria were nail psoriasis present for > 1 year; nail involvement exclusively; no use of topical or systemic treatment for psoriasis in the 3 months preceding the study; and failure of the disease to respond to conventional treatments and/or severe relapse after their discontinuation. Exclusion criteria were pregnancy and lactation; debilitating haematological, hepatic, infectious, neoplastic or neurological conditions; and immunodepression of any kind.

In total, 12 adult patients (8 women, 4 men, age range 35–56 years) with biopsy‐proven nail‐matrix psoriasis limited to 3 nails, were enrolled in the study starting from January 2019, with the study ending in September 2020. All patients had comparable medical and personal history verified to avoid potential biases. Patients were divided into two groups of six patients each: Group 1 was treated with MTX 25 mg/mL and Group 2 with TA 10 mg/mL. Each group had a baseline Nail Psoriasis Severity Index (NAPSI) of 5.3.

All necessary investigations were performed before and at each visit during MTX injections and patients were asked to take folic acid 5 mg once weekly (not on the day of injection) to reduce MTX‐associated AEs and toxicities. No investigations were considered necessary for patients undergoing TA injections.

In total, 20 nails in the 12 patients were treated. Depending on the group, either MTX or TA was injected into each affected nail, following the De Berker technique^8^ and without digital anaesthesia, although this was offered to each patient. For the MTX injections, a prefilled syringe containing MTX 25 mg/mL was used, whereas for the TA injections, the solution was injected using a Luer lock syringe with a 30G 13mm needle. Both drugs were slowly injected until bleaching from fluid load was evident for TA and until a slight yellowish skin discolouration was evident for MTX. For each injection a 0.1 mL of solution was infiltrated. At baseline and at each visit, including follow‐up visits, pictures of the 20 nails were taken to monitor the course of the disease and to submit them to an external evaluator (MI) for a further blinded visual scoring. Patients were treated every 6 weeks for 24 weeks (total of four treatment sessions) and followed up for an additional  6 months. The results are reported in Table 1.

**Table 1:** Data of patients treated with intralesional injections of methotrexate or triamcinolone acetonide.

Patient and treatment	Sex	Age, y	Affected nails (body part)	Disease duration, y	NAPSI						
					BL	Treatment session				Follow‐up month	
						1	2	3	4	1	6
MTX											
1	F	38	Fourth (LH)	3	4	3	2	1	0	0	0
2	F	49	First (RH)	2	4	3	2	1	0	0	0
3	M	39	First (RH and LH)	1	8	6	4	4	1	1	1
4	F	53	First, second (RH)	3	6	4	3	2	1	1	1
5	F	38	First (RF)	3	3	2	1	1	0	0	0
6	M	48	Third, fourth, fifth (LH)	2	7	5	4	4	0	0	0
TCA											
1	F	35	First, LH	2	4	3	3	2	1	1	1
2	F	51	First, LH	2	5	4	3	3	2	2	2
3	M	44	First, second (RH)	1	7	6	4	4	2	2	2
4	F	56	First (RH and LH)	2	5	4	3	2	2	2	2
5	F	37	First (LF)	3	3	2	2	1	1	1	1
6	M	45	First, second, third (LH)	3	8	6	4	3	3	3	7

BL, baseline; LF, left foot; LH, left hand; MTX, methotrexate; NAPSI, Nail Psoriasis Severity Index; RH, right hand; TA, triamcinolone acetonide.

**Figure 1:**
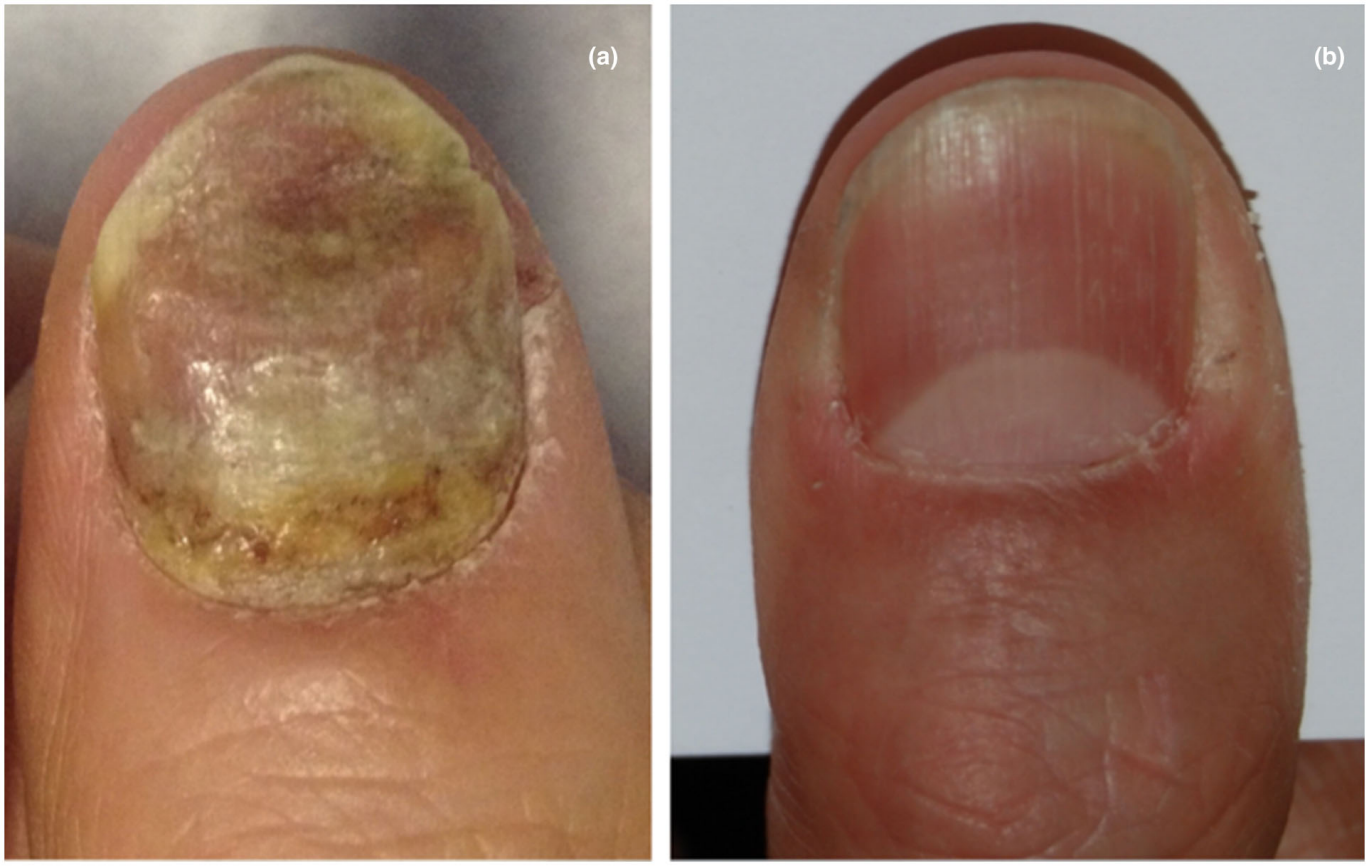
(a,b) Patient 6 (a) before and (b) after four sessions of intralesional injections of methotrexate. [Colour figure can be viewed at wileyonlinelibrary.com]

**Figure 2:**
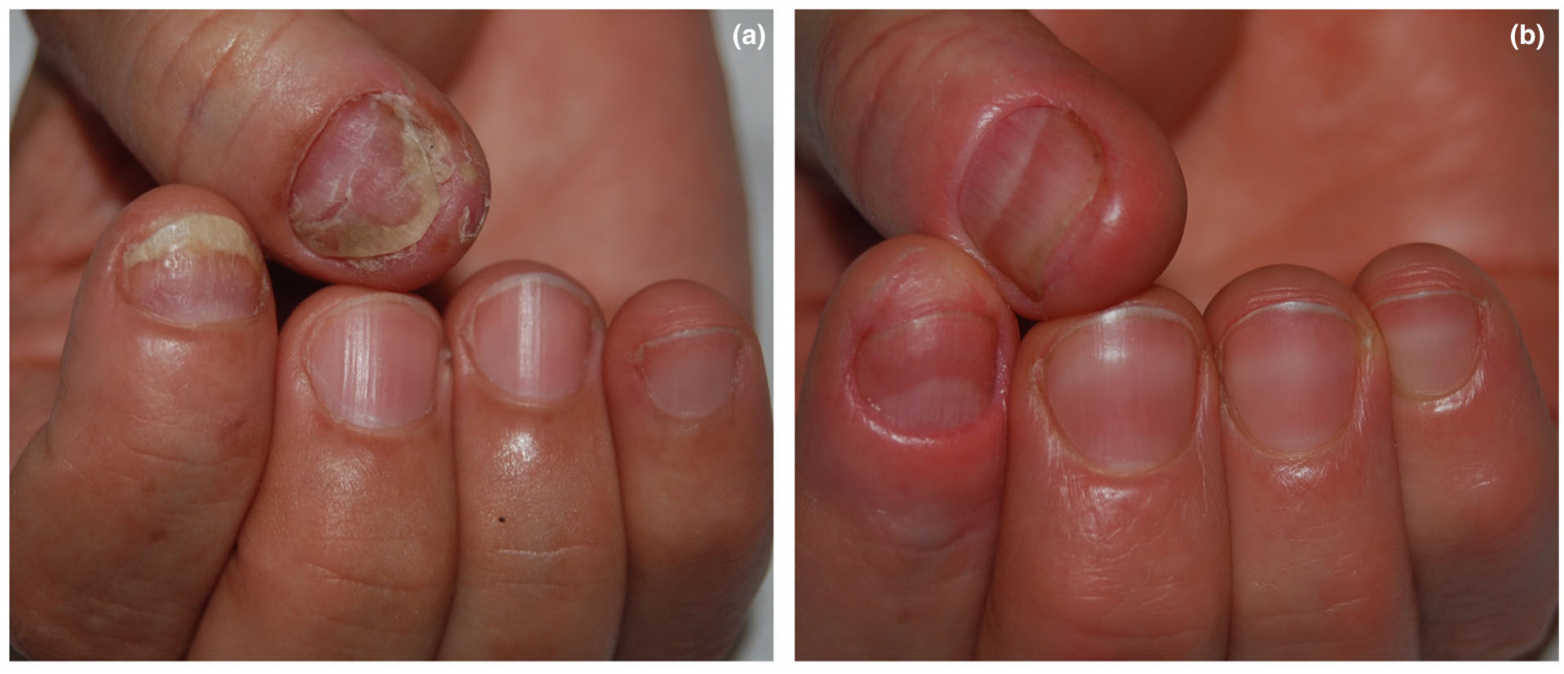
(a,b) Patient 2 before (a) and after (b) 4 sessions of intralesional injections of triamcinolone acetonide. [Colour figure can be viewed at wileyonlinelibrary.com]

At the end of the four sessions, all patients showed improvement of their nail psoriasis (Figs 1 and 2) and no new nail disease was noted: mean NAPSI at 1 month after the last treatment session had a mean value of 0.3 for the MTX group and 1.8 for the TA group. These data were confirmed at 6 months follow up for MTX, but not for TA due to worsening of the condition in one patient who reported a NAPSI 3 at 1‐month follow‐up and a NAPSI 7 at the 6‐month follow‐up.

All patients were satisfied with the procedure despite procedural pain in all of them that was, however, well tolerated. No major AEs were reported. Subungual haematoma occurred in one patient treated with MTX and in one patient treated with TA. Hypopigmentation of the proximal nail fold was instead reported in two of six patients treated with TA.

MTX, a folic acid antagonist with anti‐inflammatory and immunosuppressive properties, has been successfully used for the systemic treatment of multiple inflammatory diseases including psoriasis and psoriatic arthritis. MTX exerts its cytotoxic effects, especially on rapidly multiplying tissues with high growth fraction, inducing a reduced release of inflammatory cytokines such as tumour necrosis factor‐α, interferon‐gamma, interleukin‐12 and interleukin‐6.^9^

According to the literature systemic MTX is well known to improve nail psoriasis where mainly the matrix is affected.^10^ It needs however high doses and several months of administration to reach effectively the nail unit: at least 6–8 weeks are necessary to see the first results that become maximal after 3–6 months. Potential toxicity related to the systemic administration, moreover, can exceed the expected benefits and contraindicates treatment in case of involvement of few nails without joint/skin involvement or in the presence of comorbidities.

In the presence of nail matrix psoriasis limited to few nails, high potency steroid as clobetasol propionate 0.05% cream is a valid option,^1^ but it has been proven that if applied for a long time, it may cause bone atrophy. Intralesional TA injections are then a better solution, but MTX intralesional injections seem also to be a safe and effective alternative according to published studies and to our case series.

Larger studies are however necessary to establish the optimal dosage, number and frequency of injections to clear up the condition, and maximum duration of treatment. A long‐term follow‐up is also needed to detect possible relapses after drug withdrawal. A placebo arm should be also useful to draw definitive conclusions and to understand if improvement is due to the injected drug or to a reverse Koebner phenomenon (disappearance of a dermatosis after the site of injury).

**Learning points**

- Nail psoriasis is a difficult to treat disorder, especially in its isolated form.
- The quality of life of affected patients is markedly reduced due to the chronic course of the disease and frequent relapses.
- Intralesional TA injections are considered a safe and effective method for the treatment of nail psoriasis.
- Intralesional MTX injections have also been reported as a safe and effective treatment.
- In this study, nail‐matrix psoriasis responded very well to both treatments, but there were fewer AEs and more stability at follow‐up for the MTX‐treated patients.
- and larger studies are warranted to draw definite and statistically relevant conclusions.

## Data Availability

Data sharing not applicable to this article as no datasets were generated or analysed during the current study.
